# A reliable morphological method to assess the age of male *Anopheles gambiae*

**DOI:** 10.1186/1475-2875-5-62

**Published:** 2006-07-27

**Authors:** Bernadette J Huho, Kija R Ng'habi, Gerry F Killeen, Gamba Nkwengulila, Bart GJ Knols, Heather M Ferguson

**Affiliations:** 1 Ifakara Health Research and Development Centre, PO Box 53, Off Mlabani Passage, Ifakara, Tanzania; 2 Department of Zoology and Marine Biology, University of Dar es Salaam, PO Box 35064, Dar es Salaam, Tanzania; 3 Department of Public Health and Epidemiology, Swiss Tropical Institute, Socinstrasse 57, Basel, C4-4002, Switzerland; 4 School of Biological and Biomedical Sciences, Durham University, Durham DH1 3LE, UK; 5 International Atomic Energy Agency (IAEA), Agency's Laboratories Seibersdorf, Seibersdorf A-2444, Austria; 6 Laboratory of Entomology, Wageningen University and Research Centre, PO Box 8031, 6700 EH Wageningen, The Netherlands

## Abstract

**Background:**

Release of genetically-modified (GM) or sterile male mosquitoes for malaria control is hampered by inability to assess the age and mating history of free-living male *Anopheles*.

**Methods:**

Age and mating-related changes in the reproductive system of male *Anopheles gambiae *were quantified and used to fit predictive statistical models. These models, based on numbers of spermatocysts, relative size of sperm reservoir and presence/absence of a clear area around the accessory gland, were evaluated using an independent sample of mosquitoes whose status was blinded during the experiment.

**Results:**

The number of spermatocysts in male testes decreased with age, and the relative size of their sperm reservoir increased. The presence of a clear area around accessory glands was also linked to age and mating status. A quantitative model was able to categorize males from the blind trial into age groups of young (≤ 4 days) and old (> 4 days) with an overall efficiency of 89%. Using the parameters of this model, a simple table was compiled that can be used to predict male age. In contrast, mating history could not be reliably assessed as virgins could not be distinguished from mated males.

**Conclusion:**

Simple assessment of a few morphological traits which are easily collected in the field allows accurate age-grading of male *An. gambiae*. This simple, yet robust, model enables evaluation of demographic patterns and mortality in wild and released males in populations targeted by GM or sterile male-based control programmes.

## Background

Vector control is one of the few proven ways to reduce malaria transmission [1-7], but the effectiveness of this approach, however, is threatened by the emergence of resistance by mosquitoes to insecticides [8-10]. This phenomenon, combined with the increasing resistance of *Plasmodium *to chemotherapy [11-15], could substantially exacerbate disease prevalence, morbidity and mortality in Africa. To mitigate the consequences of resistance, new vector control interventions for reducing the malaria burden are urgently needed. One potential new tool is the genetic manipulation (GM) of *Anopheles *mosquito populations [16,17], whereby genes that prevent mosquitoes from being infected by malaria are identified and introduced into wild vector populations [18]. Ethical considerations dictate that only male mosquitoes should be used to carry these refractory genes into wild populations [19,20], as releasing females could increase biting nuisance and transmission of other *Anopheles *transmitted pathogens, including malaria if the refractory genes are not 100% efficacious.

Given the reliance of GM malaria control strategies on male *Anopheles*, the need to understand the factors that regulate their reproductive fitness, including their mating competitiveness and survival, is considerable. At present, most knowledge of male *Anopheles *survival under natural conditions comes from mark-recapture studies [21-25]. Although useful, the dispersal and low recapture of males [23-25] makes it difficult to obtain robust survival estimates from this method. A simpler alternative would be to identify traits that could be used to age-grade and assess the mating status of males on first capture, a feat which is possible with their female counterparts [26,27]. So far, there has been only two attempts to develop a morphological technique for identifying the age and mating history (mated or virgin) of male mosquitoes [28,29]. These methods were developed for the Asian malaria vectors *Anopheles culicifacies *and *Anopheles stephensi *over twenty years ago, in pioneering work by Mahmood and Reisen [28,29]. Although generally a successful technique for evaluating age in these species, the method is not widely applied and has never been evaluated for African malaria vectors in the *Anopheles gambiae *species complex.

Here for the first time, an adaptation of the age and mating status determination method developed for Asian *Anopheles *[28,29] to the African malaria vector, *An. gambiae *s.s. is presented, and its precision in predicting age and mating status of males of unknown background evaluated. The study aimed to test whether male morphological features that are easily observable under field conditions could be used to give robust estimates of male age and mating history. If successful, this methodology could be used to provide baseline measures of male *An. gambiae *fitness in the wild, based on which the relative performance of GM and/or sterile laboratory-reared mosquitoes could be monitored and compared after release.

## Methods

### Mosquito rearing

*An. gambiae s.s*. pupae were obtained from a colony maintained at the Ifakara Health Research & Development Centre (IHRDC). This colony was established in 1996 from individuals collected from Njage village in Kilombero District, Tanzania. In the insectary, pupae were collected and held individually in plastic tubes (4.9 × 2.9 cm) that were covered by netting. Pupae were left overnight for emergence, and the sex of the emerged adults identified visually the following day. Thereafter, adults of the same sex and age were pooled in groups of 50 and held in netted cages (20 × 20 × 15 cm). Mosquitoes were classified as being '0' days old on the day of their emergence. All females were at least two days old before being used in experiments described below. While in cages, mosquitoes were fed on a 10% glucose solution that was administered by placing a soaked cotton wool pad on top of the cage.

### Age and mating status determination experiments

Virgin males isolated at emergence were left for periods of 1–20 days. On each day of age, the gonads of at least 10 males were dissected and observations made as described below (following Mahmood & Reisen [28]), in order to assess how the morphology of their reproductive organs changed through time. In order to test whether morphological traits could predict whether mating had occurred, and whether any observed relationships between male age and morphology were altered by mating, experiments were conducted to provide a sample of males who had copulated before observation. On each day of mating experiments, 50 male *An. gambiae *of the same age were placed in front of a window prior to dusk (age groups ranged from 1–18 days old). Activity inside the cages was observed to begin approximately 45 minutes before dusk. Once males began to swarm, 20 virgin females (2–4 days old) were introduced into the cage. Two observers monitored activity in the cage with the assistance of a red light. When pairs of mosquitoes in copula were observed, they were siphoned from the cage and transferred together into a separate holding cup. The following morning, both males and females captured in copula were dissected. First, the spermatheca of the female was dissected to confirm whether she had been inseminated [27]. Then the male partner of the inseminated female was dissected, and the morphological features of their reproductive system compared to those of male virgins of the same age. A further sample of males caught in copula were left for a number of days after mating (2–5) before being dissected in order to estimate the duration of any observable morphological changes associated with mating. Males were not observed to mate on the day of their emergence, so these experiments were restricted to males that were 1 day post-emergence or older.

### Male dissection and morphological examination

**Figure 1:**
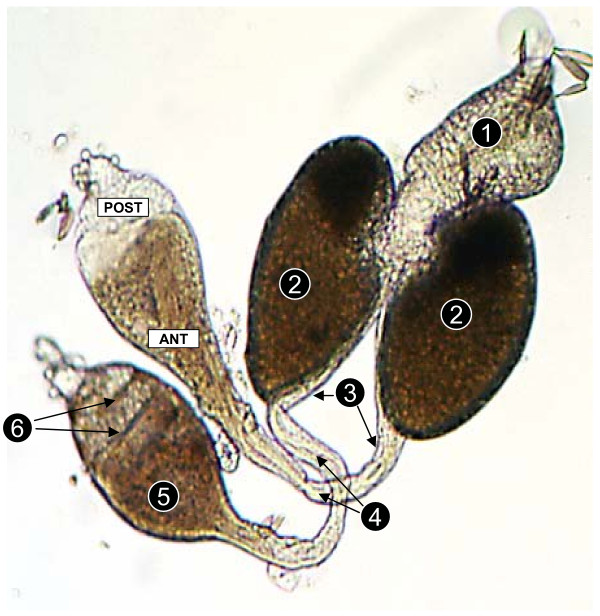
The reproductive system of male *An. gambiae *showing features used in age grading and mating status determination. The numbers on the figure represent; 1 -ejaculatory duct, 2 -accessory glands, 3 -seminal vesicle, 4 -vas efferentia 5 -sperm reservoir, and 6 – spermatocysts.

**Figure 2:**
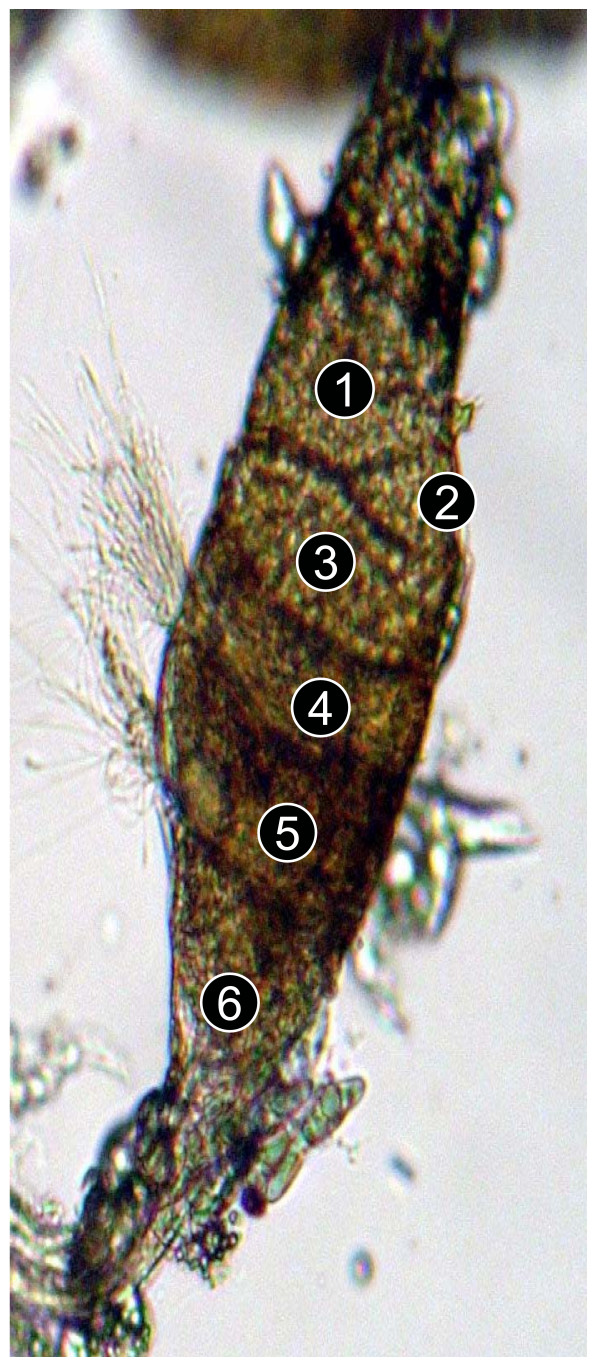
A testis of a male *An gambiae *with five spermatocysts (no. 1–5), and a developing sperm reservoir (6).

After participating in experiments, live males were incapacitated by shaking them in a holding cup. Once knocked out, they were placed in a drop of phosphate buffered saline (PBS) and examined under a dissecting microscope (50×). Their reproductive system was removed by using needles to pull out the last segment of their abdomen. Slow excision removed the whole male reproductive system including testes, accessory glands, and ejaculatory duct. Subsequent observations were made under a compound microscope (100×) as this more clearly revealed the ultrastructure of male reproductive organs; including the testes and accessory glands. Each testis holds spermatocysts that store immature spermatozoa, and a sperm reservoir that holds mature spermatozoa. Spermatocysts are visible from the posterior tip of the testis (Figure 1). As spermatocysts mature, they break down and release spermatozoa into the sperm reservoir, which occupies the anterior section of the testis (Figures 1 and 2). During copulation, spermatozoa leave the sperm reservoir and travel via the vas efferentia to the seminal vesicle and then the ejaculatory duct before entering the female. Under a compound microscope, spermatocysts appear as circular cellular structures (Figure 2) whilst the sperm reservoir is characterized by non-packaged, thread-like striations.

**Figure 3:**
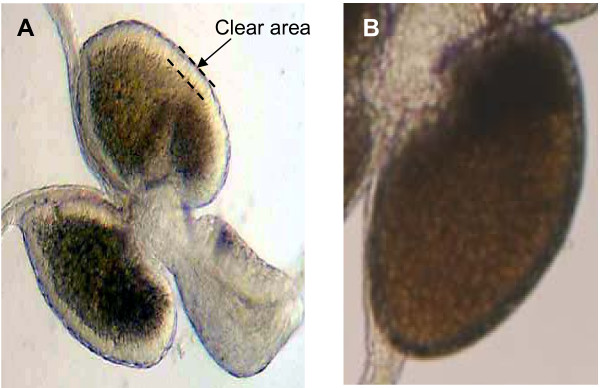
The accessory glands of a male *An. gambiae *with (a) and without (b) a clear area.

Mahmood and Reisen [28,29] demonstrated that it was possible to predict the age and mating status of *An. stephensi *and *An. culicifacies *from changes in their testicular and accessory gland morphology. This study concentrated on observation of the same traits, specifically: (1) the number of spermatocysts in the testes, (2) the proportion of testes filled by the sperm reservoir, and (3) the presence or absence of a translucent border (defined as a clear area) surrounding the accessory gland (Figure 3).

### Development of the qualitative model

Before subjecting the data to statistical analysis, some general trends between male age and their morphology were recognized. These general trends were used to develop a qualitative model for age-grading based on the values of each of the three key morphological traits (Table 1). In developing this model, the aim was to test whether male age could be predicted by following a general 'rule-of-thumb', or whether more intensive quantitative statistical analyses were required for accuracy. A qualitative model for mating status could not be developed as no clear morphological associations were observed prior to statistical analysis.

**Table 1:** Specifications of the qualitative model for age-grading, describing the required combination of morphological traits for a male to be estimated as 'young' (≤ 4 days), or 'old' (> 4 days) under this model.

Number of spermatocysts	Percentage of testis occupied by the sperm reservoir	Clear area present	Predicted age (days)
3 – 5	10 – 50%	Yes or no	≤ 4
0 – 2	50 – 100%	no	> 4

### Development of a predictive model and statistical analysis

General linear models were performed to investigate the relationship between the known age of males and: (1) the number of spermatocysts in individual testes, and (2) the proportion of the testes occupied by the sperm reservoir. Before analysis, data collected as proportions (% of testes occupied by the sperm reservoir) were logit transformed to improve their fit to a normal distribution. The additional explanatory variable of male mating status (virgin or mated the night before) was included in these models to examine whether it influenced any apparent relationship between male age and spermatocyst number, or sperm reservoir. The initial maximal statistical models for both spermatocyst number and proportional size of the sperm reservoir included the main effects of male age (days), mating status and their interaction (age × mating status). Additionally, logistic regression was used to investigate relationships between the presence of a clear area around the accessory glands and male age and mating history. The presence of a clear area was treated as a binary response variable ('0' if absent, '1' if present), with male age, mating status, and their interaction, being treated as independent explanatory variables. In all analyses, non-significant terms were sequentially eliminated to yield the minimum statistically significant model for each trait.

**Figure 4:**
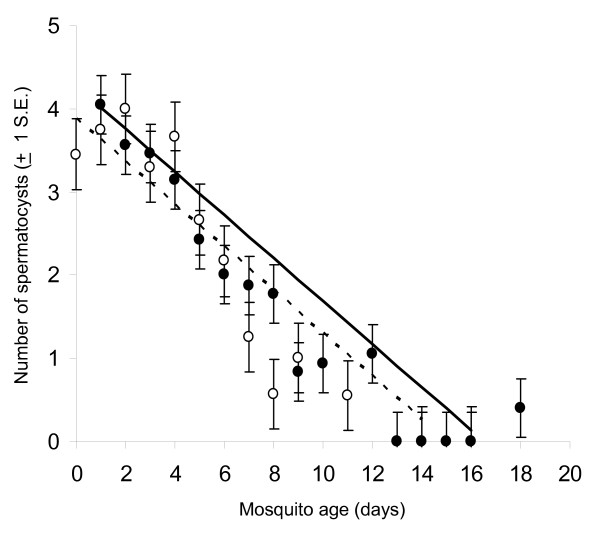
The progressive decline of spermatocyst number with age and mating in male *An. gambiae*. Lines give the best fit relationship between male age and spermatocyst number as obtained by linear regression for virgin males (dotted line and open circles) and males who had mated once (solid line and closed circles).

**Figure 5:**
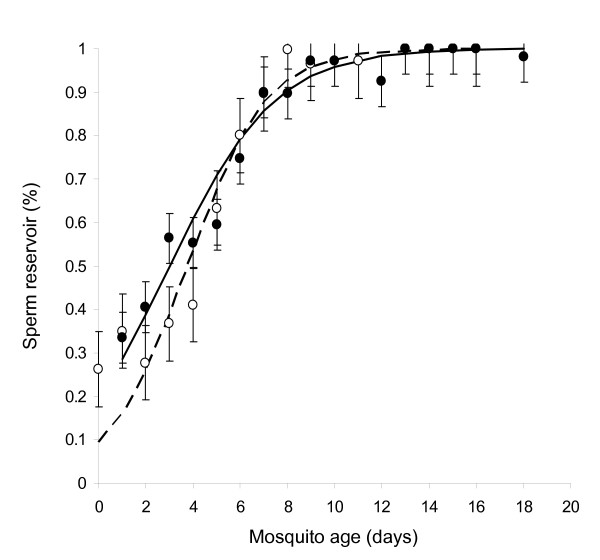
The proportion of the testis occupied by the sperm reservoir as male *An. gambiae *age. The sperm reservoir expanded faster in virgins (grey broken line and open circles) than in males who had mated once (solid black line and dark circles).

After these individual investigations of each trait, logistic regression was used to examine the ability of all three traits to predict male life-history when considered in combination. In this analysis, the outcome variable of 'age' was predicted as one of two age categories of 'young' (≤ 4 days) or 'old' (> 4 days). There are two reasons why male age was predicted as a category and not as discreet days. First, preliminary observations of the association between traits and age showed considerable overlap of values between days; suggesting a categorical rather than continuous approach could be more successful. Secondly, ideally a predictive model of male age could provide comparable demographic information as the morphology-based model available for female *An. gambiae*, that can distinguish them into groups of 'nulliparous' (assumed to be approximately ≤ 4 days old) and 'parous' (assumed to be approximately > 4 days old). By categorizing males into age groups similar to those obtained for females, this method can provide comparable estimates of survival. In this analysis, male age group was treated as a dependent variable, and spermatocyst number, proportion of testes occupied by the sperm reservoir (logit transformed) and the presence of a clear area were treated as independent variables. Binary logistic regression was used to test whether these three traits could predict whether a male was 'young' or 'old'. Four different model-fitting techniques were tested to examine which yielded the highest success in age prediction (Table 2). These models differed in whether they treated explanatory variables as continuous or categorical (spermatocyst number and relative size of the sperm reservoir), and the manner in which these explanatory variables were pooled into categories. In the first model, spermatocyst number and percent sperm reservoir were fit as continuous variables. In the following three models, values of spermatocyst number and the relative size of the sperm reservoir were pooled into categories and treated as fixed factors. In the second model, the median value of spermatocyst number (3) and the relative size of the sperm reservoir (60%) were used as cut-off points to divide data into two categories (median model). In the third, these explanatory variables were split into four categories; with the quartile values for each trait serving as cut-off points between groups (quartile model). In the final model, data for these two explanatory variables were split into four categories on the basis of visual observation for natural breakpoints in their relationships with age (Figures 4 &5, breakpoint model). In testing each of these models, all three main effects and their two-way interactions with the presence of a clear area were fit as explanatory variables. Parameter estimates obtained from the minimal statistical model for each model were used to obtain an equation for predicting age category on the basis of the observed values of each morphological feature.

**Table 2:** Description of model fitting procedures used in four quantitative statistical models of male *An. gambiae *age. In all cases, the outcome variable (y) was male age group, defined as 'young' (≤ 4 days), or 'old' (> 4 days). In each model, the relationship between three explanatory variables and the outcome variables of spermatocyst number (x_1_), proportional size of the sperm reservoir (x_2_), and the presence or absence of a clear area (x_3_) was tested. In all models, x_3 _was treated as a categorical variable, with the treatment of x_1 _and x_2 _varying between models as described below (a-d representing distinct categories).

Quantitative Model	Treatment of explanatory variables x_1 _& x_2_	No. of categories	Category definitions	
			Spermatocyst number (x_1_)	% Size of sperm reservoir (x_2_)

Continuous	Continuous	n/a	n/a	n/a
Median	Categorical	2	a = 0–2 b = 3–5	a = < 60% b = ≥ 60%
Quartile	Categorical	4	a = 0 b = 1–3 c = 4 d = 5	a = ≤ 40% b = 41 – 59% c = 60 – 94% d = ≥ 95%
Break points	Categorical	4	a = 0 b = 1–3 c = 4 d = 5	a = ≤ 30% b = 31 – 60% c = 61 – 90% d = ≥ 91%

Logistic regression was also applied to examine the association between mating status (treated as a dependent variable) and all three morphological traits. As with age, four different quantitative models were fit to explain variation in mating status (Table 2). Similar to age, all three morphological traits and their interaction were combined in a logistic regression model and used to predict the probability that a male was a virgin or had mated. This model predicted male mating status as a probability ranging between 0 and 1. Males whose mating status was predicted to be less than 0.5 were classified as virgins, and those assigned a value higher or equal to 0.5 were designated as having mated. Unless otherwise stated, error estimates accompanying means represent one standard error. All statistical analyses were performed using SAS version 8.2.

### Validation of the statistical model for age and mating status determination

In a blind trial, laboratory-reared males whose age and mating status were known only by one insectary worker were given to another researcher to dissect. The observer recorded the morphological features of these unknown males, and entered them as independent variables into the age and mating status predictive models described above. The accuracy of predictions of both age and mating status were compared to the actual values (as revealed *post hoc*). The predictions of male age obtained from the statistical model were compared with those obtained from the qualitative 'rule of thumb' (Table 1).

## Results

### Male reproductive morphology and age and mating status

**Figure 6:**
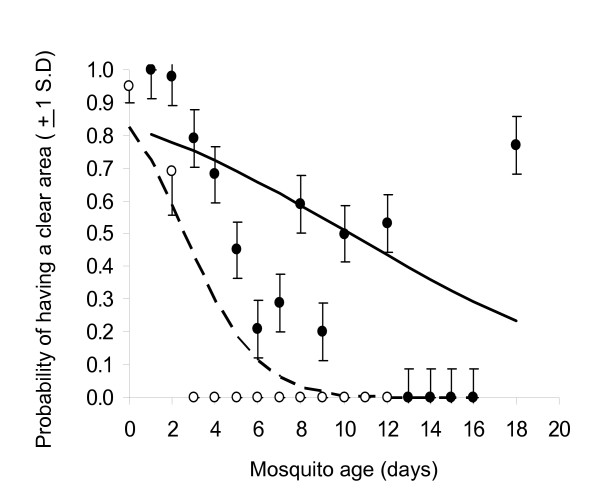
The probability of a male *An. gambiae *having a clear area around his accessory gland as a function of age and mating history. Open and closed dots are the observed frequency of having a clear area in virgin and once-mated males respectively. The broken line gives the predicted probability of virgin males having a clear area through time, and the solid line gives the predicted probability for once-mated mates.

A total of 454 *An. gambiae *males of known age were dissected. As male *An. gambiae *aged, the number of spermatocysts in their testes decreased steadily (Figure 4, F1,451 = 562.61, P < 0.01). At emergence, males had an average of 3.68 ± 0.45 (Range = 3 – 5) spermatocysts. By day 8, the mean number of spermatocysts in each testis fell below 1 (Day8 = 0.57, ± 0.57), and none were observed in unmated males in the 14 – 17 day old age group. Interestingly, a small number of spermatocysts reappeared in mated males of 18 days old (mean = 0.50, ± = 0.15). In addition to these age effects, spermatocyst number was also influenced by mating status (F 1,451= 12.09, P < 0.01), with virgin males having slightly fewer than males that had mated once (Figure 4). The rate at which spermatocysts declined with age, however, was not influenced by mating status (age × mating status: F1,450 = 2.77, P = 0.10). Conversely, the proportion of the testes occupied by the sperm reservoir increased with male age, and at a faster rate in virgins than in mated males (age × mating status: F1,450 = 45.81, P < 0.01, Figure 5). On the first day after emergence, an average of 26% (± 0.32) of the testis was occupied by the sperm reservoir. In virgin males, the sperm reservoir expanded to occupy all of the testes by day 9 of adult life, whereas once-mated males required approximately 14 days for full expansion of their sperm reservoir (Figure 5). The final morphological trait, the presence of a clear area surrounding the accessory glands, also changed with male age. The probability of having a clear area generally decreased with age, though at a substantially faster rate in virgins than in mated males (age × mating status: χ^2^_1 _= 15.85, P < 0.01, Figure 6). The majority of males in the 0–1 day old age group had a clear area surrounding their accessory glands, regardless of whether they had mated or not. By day three of adult life and onwards, none of the virgin males had a clear area, whereas some mated males still exhibited this feature up until 13 days of age. Thus males who had mated the night before dissection were more likely to have a clear area than virgins. However, this mating-related morphological change was lost through time. Sequential analysis of a cohort of 47 males who mated at 5 days of age indicated that 85% (11/13) had a clear area on the first day after mating, but only 47%, 29% and finally 0.9% displayed this trait 2, 3 and 4 days after mating respectively.

### Using morphology to predict age and mating status

To assess the ability of morphological features to predict male age grade ('young' or 'old'), all three morphological variables (logit-transformed sperm reservoir, spermatocyst number, and the presence of a clear area) and their interactions with the clear area were combined in a logistic regression model. All of the four statistical models that were fit explained a substantial proportion of the variation in male age group (r^2 ^= 0.73–0.79), and could classify the age-grade of males in the original data set with an overall accuracy of 88.1–90.5% (Table 3). Of these four, the breakpoints model had the highest predictive success when applied to the independent blind trial data, and thus was selected as the optimum model. The remainder of this section focuses on the performance of this model. Using breakpoints, the minimal statistical model of male age grade included only the main effects of the relative size of the sperm reservoir (χ^2^_1 _= 115.96, P < 0.01) and the presence of a clear area (χ^2^_1_= 33.72, P < 0.01). When fit to the original data set, this model was able to correctly categorize 89.8 % of male mosquitoes that were ≤ 4 days old, and 90.8% of mosquitoes that were > 4 days old. The accuracy of this model decreased slightly when applied to the blind trial data, although it still correctly identified 81% of males in the young age group, and 95% of those in the old group (Table 3). This quantitative model was substantially more accurate in classifying male age than was qualitative model based on generalities deduced from observation (Table 4).

**Table 3:** Comparative success of four different statistical models aiming to predict male *An. gambiae *age group as a function of their reproductive morphology (Table 2). Overall success indicates the percent of a given data set (original or blind trial) whose age-grade was correctly predicted by the model; with age-specific success indicating the proportion of 'young' and 'old' males correctly classified within these groups.

**Quantitative Model**	**Proportion of variation explained (original data)**	**Percent of data classified into the correct age groups (%)**					
		Original Data Set			Blind Trial Data Set		

	(*r*^*2*^)	Overall success	Age group	Age-specific Success	Overall Success	Age group	Age-specific success

Continuous	0.78	90.5	≤ 4 > 4	90.7 90.3	76	≤ 4 > 4	60 89
Median	0.73	88.1	≤ 4 > 4	92.6 84.0	81	≤ 4 > 4	66 93
Quartile	0.79	90.3	≤ 4 > 4	89.8 90.8	86	≤ 4 > 4	74 95
Break points	0.78	90.3	≤ 4 > 4	89.8 90.8	89	≤ 4 > 4	81 95

**Table 4:** Comparative accuracy with which the qualitative (Table 1) and quantitative (Table 2, breakpoints) models predicted the age-grade of male *An. gambiae *examined in a blind trial.

Actual age group (days)	N	Proportion of correct classification	
		Quantitative model	Qualitative model

≤ 4	73	81%	52%
> 4	88	95%	66%
Overall	161	89%	60%

The accuracy with which statistical models based on morphological features (Table 2) could correctly predict the mating status of mosquitoes in the original and blind trial was also examined. In contrast to the analysis of age, these statistical models could explain only a small proportion of the variation in male mating history (r^2 ^= 0.13–0.17, P < 0.05 in all cases, Table 5). All models suffered from the same failing; an inability to correctly identify virgin males (81–100% failure rate). Indeed, the median, quartile and breakpoints models were unable to correctly identify any virgin males, and the best performing model of mating history (continuous model) could only identify 18% of them. In this model, mating status was significantly related to the presence of a clear area (χ^2^_1 _= 21.36, P = 0.01), the proportion of the testes occupied by the sperm reservoir (χ^2^_1 _= 8.08, P < 0.04), and the interaction between the presence of a clear area and the relative size of the sperm reservoir (χ^2^_1 _= 9.81, P < 0.01). Despite the statistical significance of these terms, their ability of predict male mating success was very weak.

**Table 5:** Comparative success of four different statistical models aiming to predict the mating status of male *An. gambiae *as a function of their reproductive morphology (as described in Table 2). Mating-specific success indicates the proportion of virgin (0) and once-mated males (1) that were correctly classified by each model.

**Quantitative Model**	**Proportion of variation explained (original data)**	**Percent of data classified into the correct mating status (%)**			
		Original Data Set		Blind Trial Data Set	

	(*r*^*2*^)	Mating status	Mating-status specific success	Mating status	Mating-status specific success

Continuous	0.17	0 1	18.2 93.2	0 1	0.5 100
Median	0.16	0 1	0 100	0 1	0 100
Quartile	0.13	0 1	0 100	0 1	0 100
Break points	0.13	0 1	0 100	0 1	0 100

## Discussion

Here it has been shown that morphological traits that are easily observable under a standard compound microscope can be used to predict the approximate age of male *An. gambiae *mosquitoes with an accuracy of greater than 80%. This successful age-grading predictive model was obtained from statistical analysis of the relationships between male morphological traits and male age. However, one need not be an expert in these quantitative techniques to apply this model in the field. Of the three reproductive traits examined, knowledge of only two (relative size of the sperm reservoir and presence/absence of a clear area) was required in the optimally-performing predictive model (breakpoints). Using parameters from this model, it is possible to compile a simple table giving the predicted age for males with particular combinations of these two morphological traits (Table 6). Researchers could easily use this table in the field to obtain on-the-spot estimates of male age distribution. Thus this method can provide rapid estimates of male survival that are of relatively similar accuracy but lower cost than alternatives requiring intensive laboratory analyses (e.g. hydrocarbon analysis, [30]). Furthermore, this method generates binary age predictions that will provide comparable estimates of survival as are obtainable from morphological observation of their disease-transmitting female counterparts [26,31]. Approximations of female anopheline survival based on binary age category classification (e.g. nulliparous/parous [32-37]) have proved sufficiently accurate to monitor the impact of various control methods on female survival and population dynamics [38,39], and to explain variation in malaria transmission rates [40]. Thus, the method developed here for males could be equally capable of tracking the influence of control interventions on male *An. gambiae *demographics.

**Table 6:** Predictions of male *An. gambiae *age-grade as a function of their morphology, as obtained from the optimally performing breakpoints model. Text indicates whether males of a particular trait combination would be classified as being ≤ 4 days old (YOUNG), or older (OLD). Numbers in parentheses give the probability of males with a particular trait combination being in the 'OLD' age group, with the model categorizing all males with a probability of lower < 0.5 as being 'YOUNG', and 0.5 ≥ as being 'OLD'. Following this classification guide, the age status of 'YOUNG' and 'OLD' male *An. gambiae *should be correctly predicted on 81% and 95% of occasions respectively.

**Proportion of testis occupied by sperm reservoir**	**Age class as predicted by the optimal model (BREAKPOINTS)**	
	**Clear area absent**	**Clear area present**
**≤ 30%**	YOUNG (0.26)	YOUNG (0.04)
**31%–60%**	YOUNG (0.31)	YOUNG (0.05)
**61%–90%**	OLD (0.93)	OLD (0.62)
**> 91%**	OLD (0.98)	OLD (0.98)

Mahmood and Reisen were able to use these same morphological features to age-grade male *An culicifacies *[29]. Interestingly, the overall success rate of their morphological age-grading method when applied to *An. stephensi *was identical to that which has been obtained for *An. gambiae *(89% when applied to blind trial data in both cases, [28]). This similarity in accuracy of age prediction between this model and that of Mahmood and Reisen suggests these male mosquito reproductive traits are broadly indicative of age; both across *Anopheline *species and geographic locations. Investigation of the utility of these traits for age-grading other mosquito genera (e.g Aedes and Culicine) would be of great use to evaluate the overall generality of this approach.

In contrast to the success of the age-grading model, attempts to predict male *An gambiae *mating history based on these morphological traits failed. Both when applied to the original and blind trial data set, the statistical model, failed to identify virgins (Table 5). This bias was substantial, with 82–99% of virgins being incorrectly classified as mated. Although all three selected morphological traits changed with mating, the effect was relatively small compared to those caused by age (Figures 4, 5, 6). Thus without *a priori *knowledge of male age (precise to the day), it is unlikely that these morphological traits alone can infer whether a male *An gambiae *has mated or not. Thus additional morphological, physiological and/or behavioural traits that are more tightly linked to mating must be identified in order to reliably assess the mating history of male *An gambiae *from field samples.

Before testing quantitative statistical models for age and mating status determination, attempts to identify general 'rules of thumb' that could be followed to directly predict male age without statistical analysis were made. However, the statistical approach was substantially more robust than the qualitative 'rule-of-thumb' model discerned from early observation (89% vs. 60% overall success rate). Qualitatively, the only morphological trait linked to mating history was whether a male had a clear area around his accessory glands or not. It was noticed that male *An. gambiae *who had mated were more likely to have a clear halo around their accessory gland than those who had not, an observation also shared by Mahmood and Reisen that supports the conclusion that mating depletes accessory gland fluid [28]. Dissection of a cohort of males suggested that although mating prompts the appearance of a clear area, this feature is lost within 3–4 days of mating. This observation is also consistent with Mahmood and Reisen's investigation of *An stephensi *and *An culicifacies *[28], and confirms the notion that this feature is a transient indicator of mating, and highly confounded by age. Thus, no simple means to assess male *An. gambiae *mating history on the basis of morphology equivalent to that which is available for females was found.

In developing these age-grading and mating status models, much was learned about the basic biology and development of the male *An. gambiae *reproductive system. Similar to Mahmood and Reisen [28,29], it was established that the number of spermatocysts in male testes fell as males grew older, and that on average virgin males had slightly fewer spermatocysts than those allowed to mate once (Figure 4). As expected, the decrease in spermatocyst numbers was met by a corresponding increase in proportion of the testes occupied by the sperm reservoir (Figure 5). These changes reflect the rate of reproductive maturation of *An. gambiae *males as influenced by mating and age, with the sperm reservoir expanding as spermatocysts gradually break down and release spermatozoa into the anterior end of the testis. Working on *An. culicifacies*, Mahmood and Reisen [29] found that spermatozoa (and thus size of the sperm reservoir) and accessory gland substances were also depleted with mating. Here it was found that virgin *An. gambiae *expanded their sperm reservoir faster than mated males; suggesting that mating may deplete spermatozoa in this species also. The depletion of sperm through mating may prompt the testis to produce and mature their spermatocysts, a phenomenon that would explain why mated males of the same age had a slightly higher number of spermatocysts than virgins.

## Conclusion

Based on these findings, it is concluded that this quantitative statistical model can serve as an excellent tool for field biologists to age-grade free-living male *An. gambiae*, and be used to characterize the average survival of males from different populations. Furthermore, this model is simpler, quicker and substantially less costly to apply than some of the intensive laboratory methods currently under development. However, caution should be taken when applying this model, as researchers must be aware that it identifies 'young' males (< 4 days) with slightly lower accuracy than those who are 'old' (> 4 days, 81% vs. 95% success respectively). Thus the model may modestly overestimate the proportion of the male population in the 'old' class; and thus the survival profile of a population. Attempts to distinguish between males who had mated and males who had not on the basis of morphology were largely unsuccessful. Hopefully, other morphological or physiological traits that are more reliably linked to mating history can be identified, and used to track the mating success of genetically modified or sterile males if released into the wild. Field studies are currently underway to evaluate the utility of this age-grading method during routine mosquito surveillance. If successful, it is hoped that vector biologists working on *An. gambiae *in the wild will adopt this method widely to increase the range of tools at their disposal for understanding and reducing the expansion of this deadly disease vector.

## Authors' contributions

BH carried out the experimental work, analysis and drafted the manuscript. HF guided and advised study design, data analysis, interpretation and manuscript preparation. GK also advised on data analysis. KN assisted with the experimental work. GK, BK and GN advised on study design and manuscript preparation. All authors read and approved the final manuscript.
